# PP32 Health Technology Assessment Regulation In Europe: Implications For A National Authority

**DOI:** 10.1017/S0266462324002058

**Published:** 2025-01-07

**Authors:** Cara Usher, Emer Fogarty, Michael Barry, Roisin Adams

## Abstract

**Introduction:**

The Regulation (EU) 2021/2282 on health technology assessment (HTAR) for medicines will come into effect in January 2025; initially, new oncology medicines and advanced therapy medicinal products will be assessed at EU level. How will this work in practice? What does this mean for national HTA bodies, such as the National Centre for Pharmacoeconomics (NCPE) Ireland that uses a cost-effectiveness framework to inform decision-making for medicines?

**Methods:**

Joint work to be conducted under the Regulation includes joint clinical assessments (JCA), joint scientific consultations (JSC), and production of procedures and methodological guidance. A review was undertaken of key areas that will be impacted by the HTAR in the Irish HTA process for medicines, including timing, evidence synthesis structure, capacity building, and resource implications.

**Results:**

The HTAR will alter the current process for medicines assessment in Ireland, from early scientific advice to cost-effectiveness assessment post-authorization. JSCs will represent an additional step. Significant training and capacity implications are associated with the JCA, which will require earlier engagement with stakeholders. The NCPE’s pragmatic HTA early triaging process, the “Rapid Review,” may be delayed due to the non-duplication clause in the HTAR. The availability of high-quality comparative effectiveness evidence may help avoid full HTAs in some cases. The benefit of the JCA will be realized if the results can directly inform treatment-effectiveness estimates in cost-effectiveness modeling.

**Conclusions:**

The HTAR will significantly impact on medicines reimbursement procedures in Ireland. For the HTAR to be effective in achieving its aims, sufficient resources will need to be built into the EU HTA system. The balance between the extra resources needed and the resources spared will depend on the quality of the comparative effectiveness evidence available for the JCA.

